# Human indels as predictors of antibody responses to COVID-19 vaccines

**DOI:** 10.1016/j.isci.2025.113475

**Published:** 2025-09-01

**Authors:** Hsiuyi V. Chen, Siew-Wai Fong, Yun Shan Goh, Matthew Zirui Tay, Angeline Rouers, Zi Wei Chang, Andrea Wei Ming Chua, Liang Hui Loo, SCOPE cohort Study Group, Jean-Marc Chavatte, Raymond Tzer Pin Lin, Yee-Sin Leo, Chiea Chuen Khor, David C. Lye, Laurent Renia, Barnaby Edward Young, Lisa F.P. Ng

**Affiliations:** 1 A∗STAR Infectious Diseases Labs (A∗IDL), Agency for Science, Technology and Research (A∗STAR), Singapore, Singapore; 2 National Centre for Infectious Diseases, Singapore, Singapore; 3 National Public Health Laboratory, Singapore, Singapore; 4 Department of Microbiology and Immunology, Yong Loo Lin School of Medicine, National University of Singapore, Singapore, Singapore; 5 Lee Kong Chian School of Medicine, Nanyang Technological University, Singapore, Singapore; 6 Saw Swee Hock School of Public Health, National University of Singapore, Singapore, Singapore; 7 Tan Tock Seng Hospital, Singapore, Singapore; 8 Genome Institute of Singapore, Agency for Science, Technology and Research (A∗STAR), Singapore, Singapore; 9 Department of Medicine, Yong Loo Lin School of Medicine, National University of Singapore, Singapore, Singapore; 10 School of Biological Sciences, Nanyang Technological University, Singapore, Singapore; 11 Department of Biochemistry, Yong Loo Lin School of Medicine, National University of Singapore, Singapore, Singapore

**Keywords:** Virology, Public health

## Abstract

Vaccine efficacy varies significantly among adults. This variability underlies the limitation of a one-size-fits-all vaccination strategy and the need for more personalized approaches. We investigated factors influencing inter-individual variability in antibody responses to COVID-19 mRNA vaccine among adults. Neutralizing antibody (nAb) levels after the first vaccine dose were associated with infection outcomes within 1 year after vaccination, suggesting their potential as a correlate of protection. Age, sex, and Chinese ethnicity were associated with nAb and anti-spike protein antibody levels. Two indels located at chr1:31433042 and chr15:76311269 showed significant association with antibody responses. Leveraging these host factors, we developed a Random Forest model that predicted vaccine-induced antibody responses with 72.7% accuracy for mRNA vaccine and 76.9% for the Sinopharm COVID-19 inactivated virus vaccine. These findings support predictive modeling as a tool to identify individuals at risk of low vaccine responses, enabling more targeted and effective vaccination strategies.

## Highlights

• Vaccine responses vary, highlighting the need for personalized strategies
• Early antibody levels were predictive of infection risk post vaccination
• Age, sex, ethnicity, and two indels were associated with antibody responses
• Machine learning models predicted vaccine responses with >70% accuracy

## Graphical abstract

**Figure:**
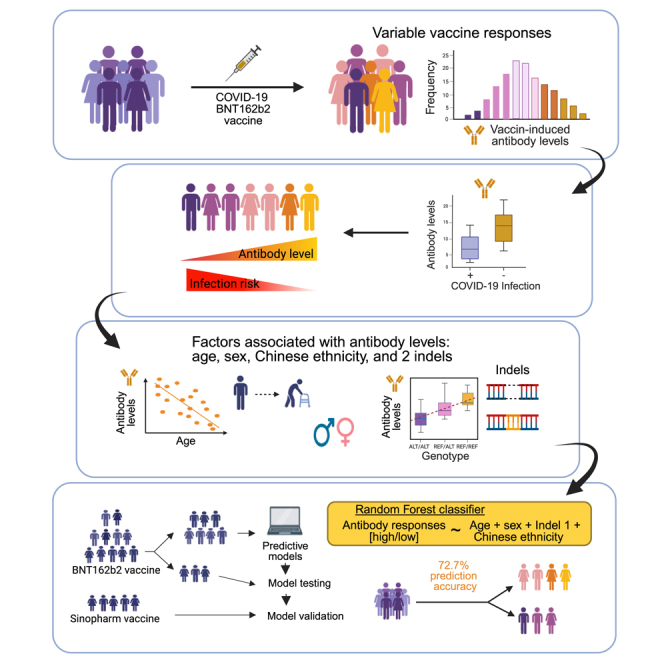

## Introduction

Substantial variations in efficacy among individuals have been observed for many vaccines.^1^^,^^2^^,^^3^^,^^4^^,^^5^^,^^6^^,^^7^^,^^8^^,^^9^^,^^10^ For example, antibody responses to vaccines for yellow fever, COVID-19, and hepatitis B vary more than 10-fold between individuals.^2^^,^^11^^,^^12^ This variability presents challenges at multiple levels. At the personal level, individuals often lack an awareness of their own response to vaccines, and those with poor responses continue to be susceptible to infections. For vaccine manufacturers, the heterogeneous responses observed during clinical trials complicate the data on vaccine efficacy. Therefore, a deeper understanding of factors that drive variations in vaccine responses could facilitate vaccine development. Moreover, by implementing more personalized strategies, identifying high-risk groups using these factors, and tailoring vaccination programs accordingly, vaccine efficacy could be improved, and disparities in vaccine protection across different populations could be reduced.

To address these challenges, we employed the COVID-19 BNT162b2 mRNA vaccine in a proof-of-concept study. Our focus was on identifying intrinsic host factors, particularly genetic variants, that influence antibody responses to vaccination. Previous studies have shown that human leukocyte antigen variants are associated with COVID-19 vaccine antibody responses.^13^^,^^14^^,^^15^^,^^16^ Beyond the genetic factors explored in this study, other variables, such as pre-existing conditions and diseases, can also influence antibody responses to vaccination. Prior research has reported that conditions like celiac disease, diabetes mellitus, chronic renal failure requiring hemodialysis, and chronic liver failure can impact antibody responses to certain vaccines.^1^ Furthermore, vaccine responses in transplant recipients may be modulated by the immunosuppressive regimens they receive.^17^ Our investigation discovered two indels, along with age, sex, and Chinese ethnicity, were significantly associated with vaccine-induced antibody responses. We developed Random Forest models for predicting vaccine-induced antibody responses using age, sex, Chinese ethnicity, and two indels as predictors. The predictive model exhibited promising performance. Our study suggests that predictive modeling can identify high-risk groups with low vaccine responses.^18^^,^^19^^,^^20^ This approach has the potential to enhance vaccination strategies by facilitating more personalized approaches, such as tailored dosing or booster schedules, that optimize vaccine efficacy and improve individual and public health outcomes.

## Results

### Variation in antibody responses to vaccines

**Figure 1:**
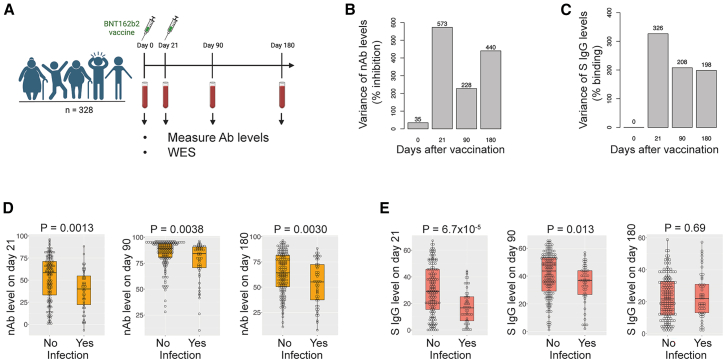
Variation in vaccine-induced antibody responses among individuals (A) Illustration of the study design: 328 individuals received two doses of BNT162b2 vaccines on days 0 and 21. Blood samples were collected on days 0, 21, 90, and 180 to measure their nAb or anti-spike protein IgG levels. (B) Comparison of variance in nAb levels on days 0, 21, 90, and 180 (% inhibition). (C) Comparison of variance in anti-spike protein IgG levels on days 0, 21, 90, and 180 (% binding). (D) Associations between infection outcomes within 1 year of vaccination and nAb levels on days 21, 90, and 180 (logistic regression, *p* value = 0.0013, 0.0038, and 0.0030 for days 21, 90, and 180, respectively). (E) Associations between infection outcomes within 1 year of vaccination and anti-spike protein IgG levels on days 21, 90, and 180 (logistic regression, *p* value = 6.7 × 10^−5^, 0.013, and 0.69 for days 21, 90, and 180, respectively). For (D) and (E), the boxplots show the median and interquartile range of the data. Ab, antibody; WES, whole-exome sequencing; nAb, neutralizing antibody; S IgG, anti-SARS-CoV-2 spike protein IgG.

The vaccine cohort in our study comprised 328 individuals who received the COVID-19 BNT162b2 mRNA vaccine, with the first dose administered on day 0 and the second dose on day 21^11^ (see also Table S1, BNT162b2 cohort). Blood samples were collected pre-vaccination and on days 21, 90, and 180 after the first dose (Figure 1A). Prior to vaccination, all participants had no known SARS-CoV-2 infections and were all negative for antibodies against the N protein when tested using the Roche N serology commercial assay.^11^ To assess the participants’ vaccine-induced antibody responses, we employed two different measurement tools. We quantified neutralizing antibody (nAb) levels using a surrogate virus neutralization test (SVNT) against the Wuhan strain,^21^ and we measured anti-SARS-CoV-2 spike protein antibody (S protein IgG) levels via a flow cytometry-based assay.^22^ These results allowed us to characterize the dynamic antibody responses that followed vaccination.

We first analyzed the variability in nAb levels across individuals (see also Table S1, BNT162b2 antibody responses). The nAb levels exhibited substantial variation after the first vaccine dose, resulting in a wide range of nAb levels on day 21.^11^ Following the second vaccine dose, 80% of participants displayed elevated nAb levels, surpassing the suggested protective threshold by day 90.^11^^,^^23^ The decline in nAb levels from day 90 to day 180 also demonstrated considerable inter-individual diversity, leading to a broad range of nAb levels on day 180. We calculated the variance in nAb levels to quantitatively assess the variation in nAb levels across the four time points. As expected, the greatest variance was observed on day 21, followed by day 180 and day 90 (Figure 1B). Similarly, considerable variations in S protein IgG levels were observed on days 21, 90, and 180, with the greatest variance in S protein IgG levels occurring on day 21 (Figure 1C). These findings highlight the pronounced variability in antibody responses following vaccination.

We then investigated whether nAb levels on day 21 could serve as a correlate of protection.^6^^,^^24^^,^^25^^,^^26^^,^^27^^,^^28^^,^^29^ After analyzing the infection records within 1 year of vaccination for 201 individuals in the cohort (who had received three COVID-19 vaccine doses in 1 year; see also Table S1, infection records), we found a significant association: individuals who had infections had lower nAb levels on day 21 compared to those who had no infections (logistic regression, *p* value = 0.0013, Figure 1D). Similar associations were found for nAb levels on day 90 and day 180, with higher *p* values than the *p* value for day 21 (logistic regression, *p* value = 0.0038 and 0.0030 for day 90 and day 180, respectively). Additionally, S protein IgG levels on day 21 were associated with infection outcomes (logistic regression, *p* value = 6.7 × 10^−5^, Figure 1E). A similar association was found for S protein IgG levels on day 90 but not day 180 (logistic regression, *p* value = 0.013 and 0.69 for day 90 and 180, respectively). These results suggest that antibody responses on day 21 could serve as a correlate of protection.

### Demographic factors influencing antibody responses to vaccines

**Figure 2:**
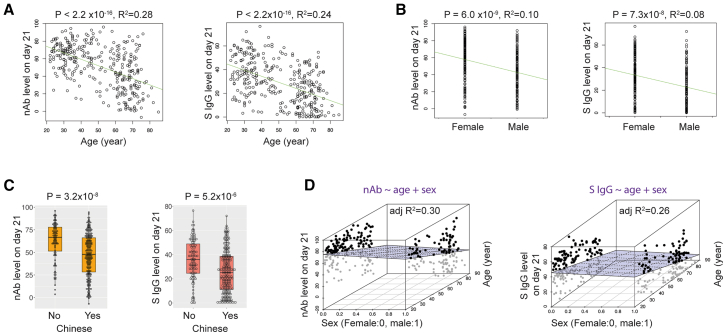
Effects of age, sex, and ethnicity on neutralizing antibody and anti-spike protein antibody responses (A) Associations between age and nAb or anti-spike protein IgG levels on day 21 (linear regression, *p* value <2.2 × 10^−16^ for both, R^2^ = 0.28 and 0.24 for nAb and anti-spike protein IgG levels, respectively, *n* = 328). Linear regression lines are plotted in green. (B) Associations between sex and nAb or anti-spike protein IgG levels on day 21 (linear regression, *p* value = 6.0 × 10^−9^ and 7.3 × 10^−8^, R^2^ = 0.10 and 0.08, respectively, *n* = 328). (C) Associations between Chinese ethnicity with nAb or anti-spike protein IgG levels on day 21 (linear regression, *p* value = 3.2 × 10^−8^ and 5.2 × 10^−6^ for nAb and anti-spike protein IgG levels, respectively, *n* = 328). The boxplots show the median and interquartile range of the data. (D) Left: distribution of nAb levels on day 21 with a multiple linear regression plane: nAb levels on day 21 ∼ age + sex, in a 3D plot (adjusted R^2^ = 0.30). Right: distribution of anti-spike protein IgG levels on day 21 with a multiple linear regression plane: anti-spike protein IgG levels on day 21 ∼ age + sex, in a 3D plot (adjusted R^2^ = 0.26). *n* = 328. nAb, neutralizing antibody; S IgG, anti-spike protein IgG.

We then aimed to identify the key determinants of antibody responses to vaccines. Specifically, we focused on nAb levels on day 21. Given that age and sex are known to affect vaccine responses,^1^^,^^6^^,^^7^^,^^9^ we first explored their associations with nAb levels on day 21. Our analysis revealed that nAb levels on day 21 were significantly associated with age (linear regression, *p* value <2.2 × 10^−16^, adjusted R^2^ = 0.28, Figure 2A) or sex (linear regression, *p* value = 6.0 × 10^−9^, adjusted R^2^ = 0.10, Figure 2B).

Ethnicity is another factor known to influence vaccine responses.^1^^,^^6^^,^^7^^,^^30^^,^^31^ Our cohort consisted of 69.82% Chinese, 10.06% Malay, 8.54% Indian, 7.93% Filipino, and 3.65% individuals from other ethnic backgrounds. These populations are relatively understudied. To delve deeper, we stratified the population into two groups: Chinese and non-Chinese. Individuals of Chinese ethnicity exhibited significantly lower nAb levels on day 21 compared to their non-Chinese counterparts (linear regression, *p* value = 3.2 × 10^−8^, Figure 2C). The association between Chinese ethnicity and lower nAb levels on day 21 remained significant after controlling for age and sex (linear regression, Chinese ethnicity *p* value = 0.0017). Similar associations were also found for S protein IgG levels of day 21 with age, sex, or Chinese ethnicity (linear regression, *p* value <2.2 × 10^−8^ for age, *p* value = 7.3 × 10^−8^, and 5.2 × 10^−6^ for sex and Chinese ethnicity, respectively; adjusted R^2^ = 0.24 and 0.08 for age and sex, respectively). Individuals in the cohort were free of autoimmune diseases, AIDS, and were not organ transplant recipients. However, some participants had reported pre-existing conditions (see also Table S1, pre-existing conditions). To avoid potential confounding effects from these pre-existing conditions on the test outcomes, we focused our linear regression analyses on participants without such conditions to examine the associations between Chinese ethnicity and antibody responses. Following adjustment for age, Chinese ethnicity showed a significant association with nAb levels on day 21 (*p* value = 0.034) but not with anti-spike protein IgG levels on day 21 (*p* value = 0.056, Figure S1).

To quantify the contribution of age, sex, and Chinese ethnicity to the variance in antibody responses to the vaccine, we performed multiple linear regression for nAb levels on day 21 using age and sex as variables. The adjusted R^2^ of 0.30 indicated that 30% of the variance could be explained by age and sex (Figure 2D). When we included Chinese ethnicity in the model, the adjusted R^2^ increased to 0.32, suggesting that all three factors combined explain 32% of the variance in nAb levels on day 21. Similar analyses for the S protein IgG levels on day 21 yielded adjusted R^2^ values of 0.26 or 0.27 for two factors (age + sex) or three factors combined (age + sex + Chinese). These findings underline the existence of additional factors influencing antibody responses to the vaccine.

### Indels associated with vaccine-induced antibody responses

Human genetic variants play pivotal roles in regulating antibody responses to vaccines.^1^^,^^32^^,^^33^^,^^34^^,^^35^ Our hypothesis posited that these genetic variants are some of the additional factors influencing vaccine-induced antibody responses. To identify such variants, we conducted whole-exome sequencing (WES) using whole-blood samples. The WES data underwent processing via GATK pipelines^36^ to detect germline small genetic variants, including indels (small insertions and deletions). Given that indels alter DNA bases in the genome, they are expected to exhibit larger effect sizes than single-nucleotide polymorphisms (SNPs). We specifically focused on indels and applied stringent filters to exclude those with low sequencing quality. Ultimately, 983 indels passed the filters, 958 of which were located in autosomes, leaving 25 indels residing on the X chromosome.

To identify indels associated with antibody responses to the vaccine, we investigated any significant association with nAb levels or S protein IgG levels on day 21. For each indel, we coded three genotypes, reference/reference (REF/REF), reference/alternative (REF/ALT), or alternative/alternative (ALT/ALT), as 0, 1, or 2. We employed an additive model for nAb level on day 21 as a function of genotype of the indel (0, 1, or 2), using age, sex, and Chinese ethnicity as covariates, to test the association (Figure S2A and see also Table S1, additiveModel nAb D21). To validate the normality assumption in the additive model, we conducted permutation tests (10,000 permutations) to obtain permutation *p* values without relying on normality assumptions. The *p* values from the permutation tests were comparable to those from the additive model, supporting the normality assumption. To address the multiple testing burden, we calculated the false discovery rate (FDR) using the Benjamini-Hochberg procedure and permutation *p* values.^37^^,^^38^^,^^39^

**Figure 3:**
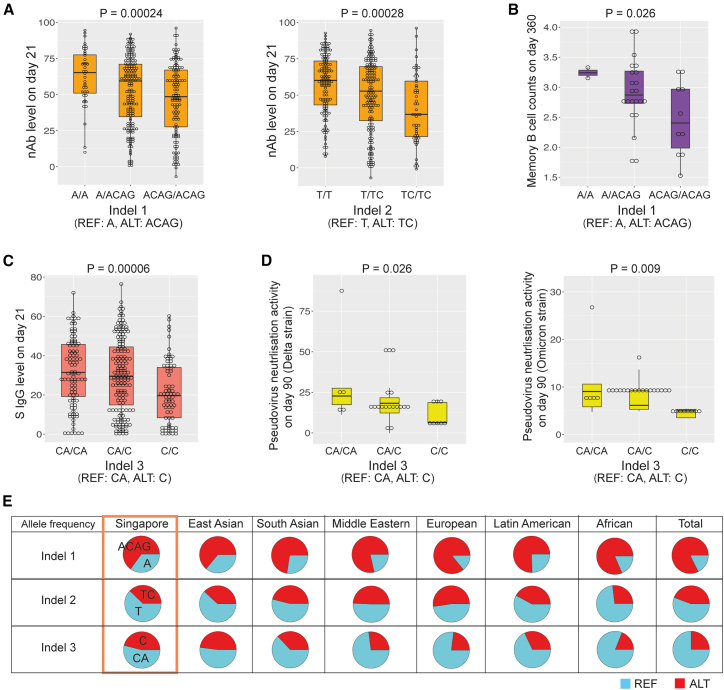
Effects of indels on antibody response to COVID-19 mRNA vaccines (A) Associations of nAb levels on day 21 with indels 1 and 2. Indel 1 REF and ALT alleles are A and ACAG; indel 2 REF and ALT alleles are T and TC. (B) Association of indel 1 with memory B cell counts on day 360 (as determined with an additive model, *p* value = 0.026). (C) Association between anti-spike protein IgG levels on day 21 and indel 3. Indel 3 REF and ALT alleles are CA and C. (D) Associations of indel 3 with pseudovirus-neutralization activities for Delta or Omicron strains on day 90 (*p* value = 0.026 or 0.009, respectively). (E) Pie charts of allele frequencies of indels 1–3 in the Singapore population in our study and different global populations in the *All of Us* program (REF allele in blue, ALT allele in red). For (A), (B), (C), and (D), the boxplots show the median and interquartile range of the data. REF, reference; ALT, alternative.

With an FDR of 0.1, we identified two indels, indel 1 and 2, which were significantly associated with nAb levels on day 21 (Table 1). Compared to the REF genotype (A), the ALT genotype (ACAG) of indel 1 was a three-base-pair (bp) DNA insertion at position chr1:31433042 in the coding region of *SERINC2*, presumably resulting in the addition of a glutamine to the serine incorporator 2 (SERINC2) protein. The ALT genotype (TC) of indel 2 was a 1-bp insertion at chr10:13142923 in a non-coding region near the optineurin gene, compared to its REF genotype (T). The ALT genotypes of both indels were associated with lower nAb levels on day 21 (Figure 3A). Further analysis revealed indel 1 to be significantly associated with reduced memory B cell counts on day 360^40^ (as determined with an additive model, *p* value = 0.026, Figure 3B, and see also Table S1, mBC data), consistent with the finding that participants who carried ACAG of indel 1 tended to have lower nAb levels on day 21.

**Table 1:** Association of indels with antibody responses induced by COVID-19 mRNA vaccine

Name	Trait	Chr	Position	Genotype		Additive model		Permutation P	FDR
				Reference	ALT	β	P		
Indel 1	nAb levels on day 21	1	31433042	A	ACAG	−6.078	2.4 × 10^−4^	2.0 × 10^−4^	0.096
Indel 2	nAb levels on day 21	10	13142923	T	TC	−5.655	2.8 × 10^−4^	2.0 × 10^−4^	0.096
Indel 3	S IgG levels on day 21	15	76311269	CA	C	−4.794	6.0 × 10^−5^	1.0 × 10^−4^	0.058

Results are shown for an additive model. β is the estimated effect size, and P is the *p* value from the additive model. Permutation P is the *p* value derived from 10,000 permutations. FDR is false discovery rate, calculated based on permutation *p* values. S IgG levels, anti-spike protein IgG levels; REF, reference allele; ALT, alternative allele.

Additionally, we discovered another indel at chr15:76311269, indel 3, that was significantly associated with S protein IgG levels on day 21 (Figure 3C and see also Table S1, additiveModel SIgG D21). Compared to its REF genotype (CA), the ALT genotype (C) of indel 3 is a 1-bp deletion in the intron of the electron transfer flavoprotein subunit alpha gene *ETFA*. Subsequent analysis revealed that the ALT genotype of indel 3 was significantly associated with pseudovirus neutralization activity against Delta or Omicron on day 90 (as determined with an additive model, *p* value = 0.026 and 0.009 for Delta and Omicron strain, respectively, Figure 3D and see also Table S1, pseudovirus data). Our analyses suggest that indel 3 may impact the expression or function of ETFA, resulting in lower S protein IgG levels and reduced neutralization activities against the virus. We validated the genotypes of indels 1–3 using Sanger sequencing for 15 individuals from the cohort. As expected, the results confirmed the genotypes of these three indels for those individuals (Figure S2B), supporting the validity of the WES analysis.

Allele frequencies of genetic variants often vary globally. Characterizing the allele frequency of a specific indel within diverse populations is crucial for estimating the global distribution of the genetic variant and identifying populations potentially susceptible to its effects. To compare the allele frequency of these indels across global populations, we leveraged data from the *All of Us* Research Program,^41^ a multi-ethnic cohort study^42^ with 245,400 participants at the time of writing, 3.03% of whom are Asian. Our Singapore cohort was also multi-ethnic but with the majority of participants being East Asian. We identified all three indels in the database of *All of Us*, confirming their presence in global populations. The alternative allele frequencies of indel 1 and 2 were comparable across different world populations (Figure 3E). By contrast, indel 3 exhibited the highest alternative allele frequency in East Asians compared with other ethnicities, suggesting that East Asians might be more susceptible to its effects. Notably, for indel 1, the ALT allele exhibited a higher allele frequency than the REF allele, highlighting that the REF allele is not invariably the major allele with highest allele frequency.

To investigate the mechanisms by which these indels affect antibody response, we developed specific hypotheses for each indel. As indel 2 resides within a non-coding region for which there is limited prior information, we focused our subsequent analyses on indel 1 and indel 3. Indel 1 is a CAG insertion in exon 10 of *SERINC2* gene, which presumably introduces an additional glutamine residue (Q) into a five-glutamine cluster (QQQQQ). We observed that the ALT allele of indel 1, which is the major allele, exhibiting frequencies of 0.64 in Each Asians and 0.86 in Europeans, is associated with lower nAb levels. Four additional insertions and one SNP at the same locus have been reported in *All of Us* and *UK Biobank* (see also Table S1, allele frequency of indel 1), but unlike indel 1, these variants have allele frequency below 0.0002. This suggests that insertions resulting in a seventh or eighth glutamine residue, or a histamine residue, are rare and potentially detrimental. Furthermore, the five-glutamine cluster sequence is highly conserved across primates (Figures S3A–S3C). Specifically, chimpanzees, bonobos, gorillas, and orangutans all possess the ALT allele of indel 1, indicating that the sixth glutamine is not unique to humans and is likely functionally important.

However, the functional role of SERINC2 in immune cells remains largely unexplored. *SERINC2* is expressed across multiple tissues and cell types.^43^^,^^44^ Single-cell RNA sequencing (scRNA-seq) data from peripheral blood mononuclear cells (PBMCs) revealed higher *SERINC2* expression in plasmacytoid dendritic cells (pDCs) compared to other immune cell types.^43^^,^^45^ pDCs, a specialized subset of dendritic cells, rapidly produce large quantities of type I interferon (IFN) upon viral infection.^46^ They play crucial roles in promoting B cell proliferation and differentiation through IFN-α secretion and cell-to-cell contact.^46^^,^^47^^,^^48^ Notably, evidence indicates a significant increase in IFN-α-positive pDCs in healthy individuals following the first dose of the COVID-19 BNT162b2 mRNA vaccine.^49^ GTEx analysis identified indel 1 as an eQTL (expression quantitative trait locus) for *SERINC2* in multiple tissues, including lung and esophagus,^50^ suggesting a potential regulatory role for indel 1. Given our observation that indel 1 is associated with nAb levels and memory B cell counts 1 year post vaccination, we hypothesize that indel 1 influences *SERINC2* expression in pDCs, thereby modulating pDC function following vaccination.

To test this hypothesis, we randomly selected 24 individuals from the cohort, ensuring diverse genotypes for indel 1 while preserving the relationship between nAb levels on day 21 and indel 1 genotypes (Figure S3D). Total RNAs were isolated from frozen PBMCs, treated with DNase I, and reverse transcribed into cDNA. Quantitative PCR was then performed to measure *SERINC2* expression on day 21. Our findings indicate that individuals harboring ALT alleles of indel 1 exhibited a trend toward lower *SERINC2* polyA+ RNA transcript levels compared to those with REF alleles (Figure S3E), consistent with our hypothesis. Nevertheless, no significant association between indel 1 and *SERINC2* expression levels on day 21 was observed, potentially attributed to the restricted sample size.

Indel 3 represents a 1-bp deletion located within the first intron of the *ETFA* gene. scRNA-seq data have demonstrated the expression of *ETFA* across multiple immune cell types.^43^^,^^45^ GTEx analysis indicates that indel 3 functions as an eQTL for *ETFA* in various tissues and as a splicing quantitative trait locus (sQTL) for *ETFA* in whole blood and numerous other tissues.^50^ Eleven splicing isoforms of *ETFA* have been documented (Figure S4A). Furthermore, indel 3 is situated within a potential promoter element in immune cells, as identified by the ENCODE project (Figure S4C). Considering the stronger evidence for sQTL associations compared to eQTL associations in whole blood within the GTEx dataset, we hypothesize that indel 3 affects the splicing of *ETFA* in immune cells.

Considering the intronic location of indel 3 between exons 1 and 2 of the *ETFA* gene and the observed variability in exon 2 across eleven *ETFA* splicing isoforms (Figure S4A), we aimed to determine if indel 3 influences *ETFA* splicing. We selected five individuals per indel 3 genotype, ensuring the preservation of the indel 3-anti-spike protein IgG level relationship (Figure S4D). To evaluate splicing, we designed primer sets targeting the exon 1–2 and exon 6–7 junctions (Figure S4B), enabling the quantification of both total *ETFA* transcripts and those including exon 2. Our analysis revealed an opposing trend: compared to individuals harboring REF alleles of indel 3, individuals carrying ALT alleles exhibited higher levels of total *ETFA* polyA+ RNA but lower levels of exon 2-containing polyA+ RNA (Figure S4E), which supports the notion that indel 3 affects splicing. Nevertheless, the limited sample size likely contributed to the lack of statistically significant associations between indel 3 and the levels of *ETFA* transcripts.

### Predictions for vaccine-induced antibody responses

We aimed to develop a model to predict vaccine-induced antibody responses using the identified host factors: age, sex, Chinese ethnicity, and indels 1–3. To facilitate interpretation, we categorized nAb levels on day 21 as either “low” or “high” using an Food and Drug Administration-approved cutoff of 60% inhibition for low or high antibody responses.^51^ We employed the low/high categorized antibody responses as the response of the model and these host factors as our predictors. To construct the predictive model, we randomly divided 328 individuals into training and test sets (295 and 33 people, respectively). The performance of the predictive models was validated with the test set or the Sinopharm vaccine cohort as an independent test set (Figure S5 and see also Table S1, Sinopharm cohort). The Sinopharm vaccine cohort included 26 participants who received two doses of the Sinopharm COVID-19 vaccine, an inactivated SARS-CoV-2 virus vaccine (different from the BNT162b2 mRNA vaccine), on days 0 and 21. Their nAb levels were measured using the same SVNT assays on days 0, 21, 90, and 180 (see also Table S1, Sinopharm antibody responses).

**Figure 4:**
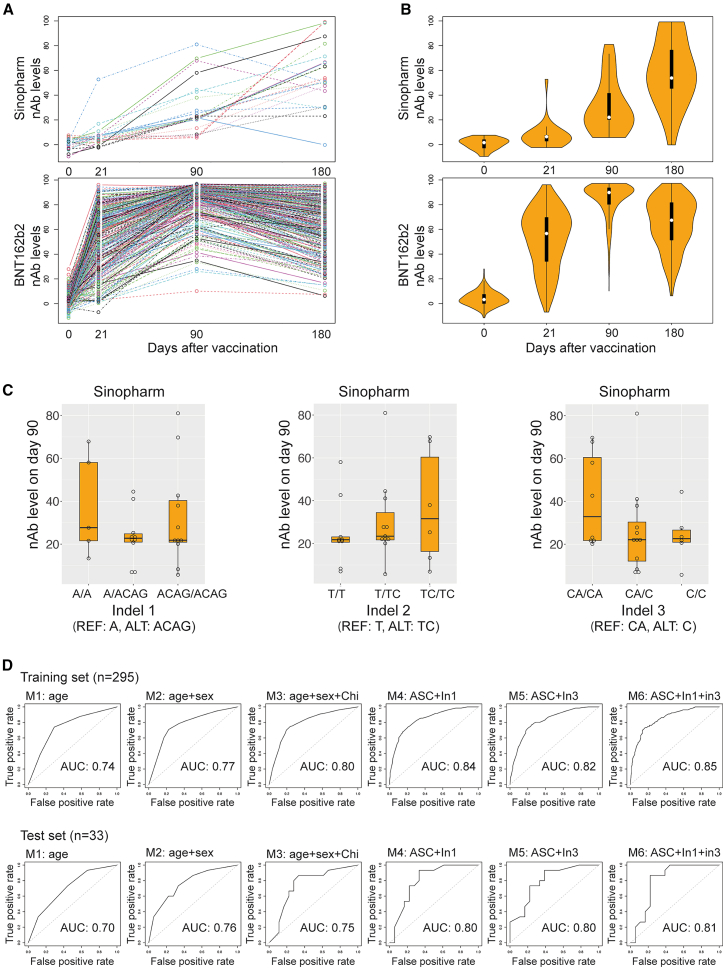
Prediction of vaccine-induced antibody responses (A) Comparison of nAb level distributions on days 0, 21, 90, and 180 in the Sinopharm inactivated virus and BNT162b2 mRNA vaccine cohorts. (B) Violin plot representation of the data shown in (A). The boxplots show the median and interquartile range of the data. (C) Effect of indel 1–3 on nAb level on day 90 in individuals in the Sinopharm cohort. No significant associations between indels and nAb level on day 90, as determined with an additive model incorporating age, sex, and Chinese ethnicity as covariates (*p* value = 0.730 for indel 1, *p* value = 0.288 for indel 2, and *p* value = 0.218 for indel 3). The boxplots show the median and interquartile range of the data. (D) Performance of Random Forest models for predicting vaccine-induced antibody responses. This panel presents receiver operating characteristic (ROC) curves and corresponding area under the curve (AUC) values for six Random Forest models. These models were trained on the BNT162b2 vaccine cohort’s training set and evaluated on its test set. Each model incorporates a different combination of variables to demonstrate the impact of additional host factors on prediction performance. The variables included are M1: age groups; M2: age groups + sex; M3: age groups + sex + Chinese ethnicity; M4: age groups + sex + Chinese ethnicity + indel 1; M5: age groups + sex + Chinese ethnicity + indel 3; M6: age groups + sex + Chinese ethnicity + indel 1 + indel 3 (Age, age groups; Chi, Chinese ethnicity; In1, indel 1; In3, indel 3; ASC, age groups + sex + Chinese ethnicity).

We compared the kinetics of the vaccine-induced nAb levels over time between the two vaccines (Figure 4A). The nAb levels induced by the Sinopharm vaccine exhibited slower kinetics compared to those induced by the BNT162b2 mRNA vaccine^52^^,^^53^^,^^54^^,^^55^^,^^56^ (Figure 4B). Thus, for the Sinopharm vaccine cohort, we focused on nAb levels on day 90 and categorized them into low/high antibody responses using the same cutoff value.

We first investigated the effect of these indels on antibody responses in the Sinopharm cohort. Our analysis revealed that for indel 1, individuals harboring ALT alleles generally displayed lower antibody responses than those with REF alleles (Figure 4C). A comparable trend was also evident for indel 3, aligning with our observations in the BNT162b2 cohort. Conversely, indel 2 exhibited an inverse trend, which diverged from the relationship seen in the BNT162b2 cohort. Notably, none of these indels exhibited a statistically significant association with nAb levels on day 90, as determined with an additive model incorporating age, sex, and Chinese ethnicity as covariates (*p* value = 0.730, 0.288, and 0.218 for indel 1, 2, and 3, respectively), possibly due to the limited sample size. Therefore, for the purpose of constructing predictive models, we selectively focused on indel 1 and 3 as potential predictor variables.

We developed Random Forest classification models by first categorizing the individuals’ ages into four groups (<34, 34–51.5, 51.5–66, and >66 years old) and establishing a base model: vaccine-induced antibody response (low/high) ∼ age groups. Subsequently, we systematically explored models by incorporating different combinations of sex, Chinese ethnicity, indel 1, and indel 3. Models were trained using the BNT162b2 cohort’s training set, with performance optimized through 10-fold cross-validation. The model demonstrating the lowest prediction error rate during cross-validation was selected, and its performance was subsequently assessed on the independent test and Sinopharm sets. The base model (model M1), which utilized age as the sole predictor, exhibited a prediction accuracy of 63.6% on the test set. To evaluate the impact of incorporating additional host factors on model predictions, we conducted a detailed precision and recall analysis, comparing the area under the receiver operating characteristic curve (AUC) performance across the training and independent test sets (Figure 4D). Our analysis of the test set performance revealed a clear pattern: the addition of sex to the base model (M1 vs. M2) increased the AUC from 0.7 to 0.76. While the further inclusion of Chinese ethnicity in model M3 did not enhance the AUC compared to M2, the subsequent addition of indel 1 (model M4) or indel 3 (model M5) to M3 improved the AUC to 0.8. Consistent with the AUC analysis, prediction accuracy increased as additional host factors were added to the base model. Model M4, which incorporated age, sex, Chinese ethnicity, and indel 1 as predictors, demonstrated the best overall performance, achieving a prediction accuracy of 72.7% on the test set and 76.9% on the Sinopharm set.

## Discussion

In this study, we identified three indels associated with vaccine-induced antibody responses, providing insights into the mechanistic pathways underlying vaccine-induced immunity. Indel 1 is characterized as a CAG insertion within the coding region of *SERINC2*. This insertion presumably introduces an additional glutamine residue into a five-glutamine cluster (QQQQQ) located in intracellular loop 4 of the SERINC2 protein.^57^ Interestingly, this insertion occurs at a homologous site in SERINC5, a proposed binding site for an acidic cluster (EDTEE) that interacts with the HIV-1 accessory protein Nef.^57^^,^^58^ While SERINC5 functions as a viral restriction factor, human SERINC2 does not exhibit such properties,^59^^,^^60^ suggesting alternative functions for SERINC2.

We found that chimpanzees, bonobos, gorillas, and orangutans also possess the ALT allele of indel 1, indicating that the presence of the sixth glutamine is not unique to humans and likely holds functional significance. Although the functional role of SERINC2 in immune cells remains largely undefined, evidence suggests that indel 1 might influence vaccine-induced antibody responses by modulating *SERINC2* expression in pDCs, thereby affecting pDC functions, including the promotion of B cell proliferation and differentiation. This hypothesis is supported by our observations of an association between indel 1 and vaccine-induced antibody responses, as well as memory B cell counts 1 year post vaccination. Furthermore, we observed a potential relationship between indel 1 and *SERINC2* expression and a similar relationship between indel 1 and vaccine-induced antibody responses in the Sinopharm cohort. Notably, the ALT allele of indel 1, associated with lower nAb levels, has frequencies of 0.64 in East Asians and 0.86 in Europeans, suggesting that this pathway could represent a potential target for adjuvant studies aimed at enhancing vaccine responses in individuals carrying ALT alleles of indel 1.

Indel 3 represents a 1-bp deletion in the first intron of *ETFA* gene. ETFA plays a crucial role in the initial stage of mitochondrial fatty acid beta-oxidation by mediating electron transfer between primary flavoprotein dehydrogenases and the membrane-bound electron transfer flavoprotein ubiquinone oxidoreductase.^61^ Mutations in the *ETFA* gene can cause multiple acyl-CoA dehydrogenation deficiency, a recessively inherited disorder of fatty acid, amino acid, and choline metabolism.^62^^,^^63^^,^^64^ Notably, indel 3 is located within a potential promoter element active in immune cells and has been identified as a sQTL for *ETFA* in whole blood based on GTEx data. Our analyses revealed that individuals carrying the ALT alleles of indel 3 presented with increased levels of total *ETFA* transcripts but decreased levels of exon2-containing transcripts, supporting indel 3’s role in *ETFA* splicing. Considering the association of the ALT allele of indel 3 with lower antibody responses, distinct genotypes of indel 3 may result in differential expression of *ETFA* splicing isoforms, thereby impacting mitochondrial function and subsequently modulating the responsiveness of immune cells to vaccination. Of particular interest is the higher ALT allele frequency of indel 3 observed in East Asian populations compared to others, which could indicate a greater potential for its effects in this population group.

While our approach for quantitative trait locus (QTL) analysis successfully identified three indels associated with vaccine-induced antibody responses, the statistical power and the precision of our estimates are potentially limited by the relatively small sample size and FDR cutoff of 0.1. Specifically, the observed effects of these indels on antibody responses within the independent Sinopharm cohort were consistent with the findings for indel 1 and indel 3 in the BNT162b2 cohort. However, the direction of effect for indel 2 did not replicate in the Sinopharm cohort. Given the limited prior knowledge regarding indel 2 and its divergent results across cohorts, we have greater confidence in the associations of indel 1 and indel 3 with vaccine-induced antibody responses. The subsequent analyses strengthen the evidence for their biological relevance in modulating the immune response to vaccination. Further investigation with larger, independent cohorts with diverse genetic backgrounds is warranted to validate these findings.

Significant associations were identified between antibody levels on day 21 post vaccination and five key host factors: age, sex, Chinese ethnicity, and two indels. The well-documented influence of age and sex on vaccine-induced antibody responses validates our study’s findings in this regard.^1^^,^^6^^,^^7^^,^^9^ Our results further indicate that individuals of Chinese ethnicity faced a higher risk of lower vaccine responses when compared to other non-Chinese Southeast Asian populations. This aligns with a recent study that reported an odds ratio of 0.5 for seropositivity to Omicron BA.1 and BA.2 strains in Chinese individuals compared to those of Malay ethnicity,^65^ suggesting a reduced likelihood of developing antibodies against these variants. It is important to consider that our cohort included a disproportionately higher number of elderly individuals within the Chinese group compared to other ethnic groups. While we accounted for age and comorbidity in our analysis, this specific finding regarding Chinese ethnicity warrants further validation in future studies with more ethnically balanced age distributions.

Our study developed predictive models for vaccine-induced antibody responses, leveraging the identified host factors of age, sex, Chinese ethnicity, indel 1, and indel 3. Given that nAb levels induced by COVID-19 mRNA vaccines are significantly associated with infection outcomes, our predictive model holds promise as a tool for assessing vaccine efficacy.^66^ Importantly, no evidence suggests that these predictors are dependent on vaccination. Consequently, our model has the potential to enable pre-vaccination predictions of vaccine efficacy.

Studies have revealed that pre-vaccination peripheral blood transcription signatures are predictive of antibody responses to vaccination.^18^^,^^19^^,^^20^ Our findings align with this prior work, demonstrating that vaccine-induced antibody responses can be forecasted before vaccination. The earlier model, utilizing a transcriptional signature of 500 genes as predictors, reported an AUC of 62.3% via 10-fold cross-validation.^18^ Our model, employing one indel and three demographic factors as predictors (distinct from those used in the earlier model), achieved an AUC of 76.9% on a small independent cohort. Notably, our model was trained using genetic backgrounds from East Asia. Despite the current population of East Asia being 20% of the global population, it remains significantly understudied. Our findings enhance the understanding of how these host factors influence vaccine responses in East Asians. Furthermore, our model’s ability to predict the response outcomes in an independent cohort that was given a different type of COVID vaccine from the training cohort with an accuracy of 76.9% suggests its potential applicability beyond COVID-19 mRNA vaccines. This hypothesis gains support from a related study that found pre-vaccination transcriptional signatures predictive of antibody responses across 13 different vaccines.^18^

However, several limitations of our current predictive model must be acknowledged. These include its suboptimal performance, the small sample sizes across the training test and Sinopharm sets, a genetic bias toward East Asian populations, and its evaluation solely using COVID-19 vaccines. A particularly notable concern is the evaluation of model performance on the Sinopharm cohort, which was based on only 26 individuals. This small sample size significantly undermines the statistical robustness and reliability of the findings, making it difficult to draw confident conclusions about the model’s effectiveness in this subgroup. To enhance the generalizability of the model, future work should involve training and testing on independent cohorts with larger sample sizes, more diverse genetic backgrounds, and data from vaccinations beyond those targeting SARS-CoV-2. This will be crucial for ultimately improving the model’s applicability across a broader range of individuals and vaccination scenarios.

In summary, our findings support the use of predictive modeling to identify individuals at high risk of low vaccine responses. This information can be leveraged to develop more effective vaccination strategies through more personalized approaches that account for individual risk factors, ultimately aiming to improve overall vaccine efficacy. Such strategies may include the provision of additional booster doses or the consideration of alternative vaccine formulations for individuals predicted to exhibit low responses. This approach holds the promise of maximize protection and minimize the risk of poor outcomes in specific populations. However, the cost-effectiveness and practical implementation of indel characterization warrant careful consideration. As the cost of DNA sequencing continues to decline, with increasing number of individuals undergoing whole-genome sequencing or WES through voluntarily initiatives (e.g., *All of Us*) and national programs (e.g., SG100K in Singapore), the cost of genotyping is likely to become manageable, thereby facilitating the practical implementation of more personalized vaccination programs. Importantly, this information should not be employed to deny vaccination but rather as a means to enhance its effectiveness through more targeted interventions.

### Limitations of the study

Several limitations of our study must be acknowledged. The QTL analysis, while identifying two indels linked to vaccine-induced antibody responses, is constrained by a small sample size and an FDR cutoff of 0.1, which may affect statistical power and precision. Our predictive model also shows suboptimal performance, limited by small cohort sizes, a genetic bias toward East Asian populations, and an evaluation restricted to COVID-19 vaccines. Notably, the Sinopharm cohort included only 26 individuals, limiting the reliability of model assessment in this subgroup. Future studies should incorporate larger, more diverse cohorts and broader vaccine types to improve model generalizability.

## Resource availability

### Lead contact

Further information and requests for resources and reagents should be directed to and will be fulfilled by the lead contact, Hsiuyi Chen (chen_hsiu_yi@a-star.edu.sg).

### Materials availability

This study did not generate new unique reagents.

### Data and code availability

• The data supporting the findings of this study are available in the supplemental table, except for the WES data. The participants did not provide written consent for their WES data to be shared publicly.
• The following software packages were used for data analysis: Burrows-Wheeler Aligner (version 0.7.17), Genome Analysis Toolkit (version 4.2.6.1), R version 4.3.1, R package ranger (version 0.16.0), and ROCR (version 1.0.11).
• Any additional information required to reanalyze the data reported in this paper is available from the lead contact upon request.

## Consortia

The members of the SCOPE cohort Study Group are Anthony Torres Ruesta, Vanessa K.X. Neo, Anna X.Y. Loo, Adeline Chiew Yen Chua, Yong Jie Tan, Pei Xiang Hor, Chiew Yee Loh, Yuling Huang, Ajayanandan Yadunandan, Sooriya Kannan Selvam, Wong Wei Lun, Peter Cheng, Jonathan Jordon Lim Caliu.

## Acknowledgments

We are grateful for the participants in the Singapore and *All of Us* cohorts for their contributions. We thank the National Center for Infectious Diseases SCOPE team in Singapore for their help in recruitment and the National Institutes of Health in US for the access to the participants’ aggregated data in *All of Us*. We also thank Patrick Tan, Wee Yang Meah, Genome Institute of Singapore (GIS) and GIS sequencing platform for their support in whole-exome sequencing, and Arko Sen for his help with data analysis. This work was supported by the A∗STAR Computational Resource Center through the use of its high performance computing facilities. The authors are grateful to the A∗STAR IDL Pathogen Flow Platform for their invaluable assistance in this study. The study was supported by the 10.13039/501100012415Biomedical Research Council (BMRC), A∗SF, A∗CRUSE (Vaccine monitoring project), the A∗ccelerate GAP-funded project (ACCL/19-GAP064-R20H-H) from Agency of Science, Technology and Research (A∗STAR), Singapore, 10.13039/501100001349National Medical Research Council COVID-19 Research Fund (COVID19RF-001, COVID19RF-007, COVID19RF-0008, COVID19RF-011, and COVID19RF-060) (L.R., L.F.P.N., D.C.L., and B.E.Y.), 10.13039/100000038US Food and Drug Administration (#75F40120C00085) (L.R. and L.F.P.N.), A∗STAR COVID-19 Research funding (H/20/04/g1/006) (L.R. and L.F.P.N.), and OF-LCG19May-0034.

## Author contributions

H.V.C. conceived the study with the help from L.F.P.N., C.C.K., S.-W.F., and L.R. L.F.P.N. supervised the study. Sample collection, J.-M.C., R.T.P.L., D.C.L., and B.E.Y. Sample processing and data collection, S.-W.F., Y.S.G., M.Z.T., A.R., Z.W.C., A.W.M.C., L.H.L., and SCOPE cohort Study Group. C.C.K. conducted WES. H.V.C. performed genetic testing, statistical analysis, and modeling. C.C.K. assisted data analysis. H.V.C. wrote the manuscript with inputs from all authors. Review and editing, all authors. Revision, H.V.C., L.H.L., S.-W.F., L.F.P.N., B.E.Y., and J.J.L.C.

## Declaration of interests

A patent application has been filed (Singapore patent#10202400609T, #10202403182P, PCT/SG2025/050121: Genetic Signatures For Predicting Vaccine Response and Uses Thereof) (H.V.C., L.F.P.N., L.R., Y.S.G., S.W.F., and M.Z.T.).

## Declaration of generative AI and AI-assisted technologies in the writing process

During the preparation of this work, the authors used copilot and Gemini in order to improve language and readability. After using these tools, the authors reviewed and edited the content as needed and take full responsibility for the content of the publication.

## STAR★Methods

### Key resources table

**Table:** 

REAGENT or RESOURCE	SOURCE	IDENTIFIER
**Chemicals, peptides, and recombinant proteins**		

Phusion high-fidelity DNA polymerase	NEB	Cat#M0530L
KAPA HyperPure beads	Roche	Cat#08963843001
DNase I	Thermo Fisher Scientific	Cat#EN0521
SsoAdvanced Universal SYBR Green Supermix	Bio-Rad	Cat#1725275

**Critical commercial assays**		

cPass™ SARS-CoV-2 Neutralization Antibody Detection Kit	GenScript	Cat #L00847-A
KAPA HiFi PCR kit	Roche	Cat #07958935001
abGenix™ whole blood genomic DNA extraction kit	AITbiotech	Cat#800815
RNeasy Mini kit	Qiagen	Cat #74106
SuperScript™ III First-Strand Synthesis System	Thermo Fisher Scientific	Cat#18080051
genomic DNA and hybridization capture kit	Roche	Roche-Nimblegen SeqCap
ELISpot Path: SARS-CoV-2 (RBD) Human IgG (ALP)	Mabtech	Cat #3850-4APW-R1-1
Monarch genomic DNA extraction kit	NEB	Cat#T3010L

**Deposited data**		

Data from surrogate virus neutralization assay, anti-spike protein antibodies by serological detection, Memory B cell ELISpot assay, and Pseudovirus neutralization assays	Renia, L., Goh, Y.S., Rouers, A., Le Bert, N., Chia, W.N., Chavatte, J.-M., Fong, S.-W., Chang, Z.W., Zhuo, N.Z., Tay, M.Z. et al. (2022). Lower vaccine-acquired immunity in the elderly population following two-dose BNT162b2 vaccination is alleviated by a third vaccine dose. Nat Commun *13*, 4615.	https://doi.org/10.1038/s41467-022-32312-1.
Human reference genome NCBI build 38, GRCh38	Genome Reference Consortium	http://www.ncbi.nlm.nih.gov/projects/genome/assembly/grc/human/

**Oligonucleotides**		

Indel1-F1: 5′-AACATCAAGGCTCATCACAGA-3′	This paper	–
Indel1-R1: 5′-GTCCTTGATACACACCGCCA-3′	This paper	–
Indel2-F1: 5′-TCTGGATGAAGATAGGCTAGGA-3′	This paper	–
Indel2-R1: 5′-GAACTTCCAGCCATTCACCA-3′	This paper	–
Indel3-F1: 5′-CAGAGAGTGGGCCAGAAGAA-3′	This paper	–
Indel3-R1: 5′-TAGTATGGACCAGGCATTGTG-3′	This paper	–
Indel3-F2: 5′-CCAAACCTCTGACTTCTGCC-3′	This paper	–
Indel3-R2: 5′-ACAGTCTGAGCTCGGTCATC-3′	This paper	–
GAPDH forward (F), 5′-CAATGACCCCTTCATTGACC-3′	This paper	–
GAPDH reverse (R), 5′-TTGATTTTGGAGGGATCTCG-3′	This paper	–
SERINC2 forward (F3), 5′-CCTATGCTAGACGCCACACA-3′	This paper	–
SERINC2 reverse (R3), 5′-TCCTGCTCGTTGTCAAAGG-3′	This paper	–
ETFA forward (F1), 5′-GTTTTCTGTCCGTGGAACATCC-3′	This paper	–
ETFA reverse (R1), 5′-ATTTCTGGTCAAGCCACTCTG-3′	This paper	–
ETFA exon 1-2 junction forward (e12juc-F1), 5′-CGTGTGGCTGCAGTAATGGT-3′	This paper	–
ETFA exon 1-2 junction reverse (e12juc-R1), 5′-GCGGCCTCATTGCTACGATT-3′	This paper	–

**Software and algorithms**		

Burrows-Wheeler Aligner (version 0.7.17)	Li H. and Durbin R. (2009) Fast and accurate short read alignment with Burrows-Wheeler Transform. Bioinformatics, 25:1754-60.	https://bio-bwa.sourceforge.net/
Genome Analysis Toolkit (version 4.2.6.1)	Van der Auwera GA & O'Connor BD. (2020). ***Genomics in the Cloud: Using Docker, GATK, and WDL in Terra (1st Edition).*** O'Reilly Media.	https://gatk.broadinstitute.org/hc/en-us/sections/5358821689883-4-2-6-1
R version 4.3.1,	*R Core Team (2023). _R: A Language and VEnvironment for Statistical Computing_. R Foundation for Statistical Computing, Vienna, Austria.*	*https://www.R-project.org*
R package ranger (version 0.17.0)	Marvin N. Wright, Andreas Ziegler (2017). ranger: A Fast Implementation of Random Forests for High Dimensional Data in C++ and R. Journal of Statistical Software, 77(1), 1–17. https://doi.org/10.18637/jss.v077.i01	https://imbs-hl.github.io/ranger/
ROCR R package (version 1.0.11)	*Sing T, Sander O, Beerenwinkel N, Lengauer T. [2005] ROCR: visualizing classifier performance in R. Bioinformatics 21(20):3940-1.*	https://www.rdocumentation.org/packages/ROCR/versions/1.0-11
Samtools	Li H., Handsaker B., Wysoker A., Fennell T., Ruan J., Homer N., Marth G., Abecasis G., Durbin R. and 1000 Genome Project Data Processing Subgroup (2009) The Sequence alignment/map (SAM) format and SAMtools. Bioinformatics, 25, 2078-9.	http://samtools.sourceforge.net/
FlowJo	Tree Star	–
pROC library	R version 3.6.4	–

### Experimental model and study participant details

A total of 328 healthcare workers and older individuals were recruited to the COVID-19 BNT162b2 mRNA vaccine cohort, while 26 adults were enrolled in the Sinopharm inactivated virus vaccine cohort (See also Table S1, BNT162b2 cohort, and Sinopharm cohort).^11^ Participants received the first dose of their respective vaccines on day 0 and the second dose on day 21. The BNT162b2 cohort included 138 male and 190 females, whereas the Sinopharm cohort comprised 11 male and 15 females. Blood samples were collected prior to vaccination (day 0) and on days 21, 90, and 180 following the first dose (Figure 1A). Detailed distributions of age, sex, ethnicity, and genotype for both cohorts are provided in Table S1. The study design and protocol received approval from the National Healthcare Group (NHG) Domain Specific Review Board (DSRB) under study number 2012/00917. Additionally, the SingHealth Centralized Institutional Review Board (CIRB) approved the collection of samples from healthy donors under study number 2017/2806 and NUS IRB 04–140. Written informed consent, in accordance with the Declaration of Helsinki for Human Research, was obtained from all participants. Inclusion criteria are 18 years of age and older. Able to read, speak, and understand English and in good general health. Individuals with a prior SARS-CoV-2 infection were excluded.

### Method details

#### Infection records

This analysis examined infection records for one year following the first vaccine dose. 204 Individuals experienced either no infection or a single infection within this period. Three individuals with infections between 179 and 183 days were excluded. Infections occurring between 200 and 365 days post-vaccination were included (Table S1). Infection data was self-reported during follow-up visits and confirmed via ART or PCR testing.

#### Whole exome sequencing

Genomic DNA was extracted from whole-blood samples of 328 individuals in the BNT162b2 cohort using an abGenix whole blood genomic DNA extraction kit (AITbiotech, #800815). Libraries for whole-exome sequencing (WES) were prepared using a genomic DNA and hybridization capture kit (Roche-Nimblegen SeqCap), and later sequenced with pair-end 150-bp reads on Illumina instruments in Genome Institute of Singapore sequencing platform (Singapore).

#### WES data processing

Each sample was sequenced, yielding approximately 45 million reads with an average coverage of 56. The reads were mapped to the human genome build GRCh38.p13 using the Burrows-Wheeler Aligner (version 0.7.17)^67^ and Samtools.^68^ The germline short variant discovery pipeline of the Genome Analysis Toolkit (version 4.2.6.1)^36^ was used to call indels. The called indels underwent stringent filtering. Exact tests of Hardy–Weinberg Equilibrium were performed to filter variants with excess heterozygosity. In addition, VariantRecalibrator, which builds a recalibration model to score variant quality, was used to filter out indels of low quality. Then, hard filters were applied to select indels (excluding multi-allelic indels) of high quality: FS = 0, SOR <0.8, MQ ≥ 60, ExcessHet <15. For any genotype of an indel, if the total number of individuals with the indel was <40, the indel was also excluded. In the end, 983 indels passed the filter stage.

#### Sanger sequencing

The same genomic DNAs used for WES were used for Sanger sequencing. For the Sinopharm cohort, genomic DNAs were extracted using Monarch genomic DNA extraction kit (NEB, T3010L). In general, primers were designed to amplify the region from 200 bp upstream of the indel to 800 bp downstream of the indel. The primers targeting indels 1–2 were as follows: Indel1-F1: 5′-AACATCAAGGCTCATCACAGA-3′, Indel1-R1: 5′-GTCCTTGATACACACCGCCA-3′. Indel2-F1: 5′-TCTGGATGAAGATAGGCTAGGA-3′, Indel2-R1: 5′-GAACTTCCAGCCATTCACCA-3′. The following two pairs of primers were used to sequence Indel 3: Indel3-F1: 5′-CAGAGAGTGGGCCAGAAGAA-3′, Indel3-R1: 5′-TAGTATGGACCAGGCATTGTG-3′; Indel3-F2: 5′-CCAAACCTCTGACTTCTGCC-3′, Indel3-R2: 5′-ACAGTCTGAGCTCGGTCATC-3’.

A 40-ng sample of genomic DNA was amplified using Phusion high-fidelity DNA polymerase (NEB, M0530L) and indel-specific primers for 30 cycles in a Bio-Rad thermocycler C1000. After confirming the PCR products to be single bands by 1% TAE gel electrophoresis, the PCR products were purified using KAPA HyperPure beads (Roche, 08963843001). The purified PCR products were sent to 1^st^ Base (Singapore) for Sanger sequencing. A KAPA HiFi PCR kit (Roche, 07958935001) was used to amplify the Indel 3 sequence for some samples due to the location of this indel within a high GC% sequence. Samples from 15 individuals from the BNT162b2 mRNA vaccine cohort and from all 26 individuals in the Sinopharm cohort were sequenced.

#### Associations between indels and antibody responses

Of the 983 indels that passed the filter stage, 25 were located on the X chromosome. For the 958 autosomal indels, the genotypes of each indel were coded as 0 for REF/REF, 1 for REF/ALT, and 2 for ALT/ALT. Association of an indel with nAb levels or anti-spike protein IgG levels on day 21 was tested using the additive model: nAb levels on day 21 ∼ β_0_ + β_1_∗age + β_2_∗sex + β_3_∗Chinese ethnicity + β_4_∗genotype of Indel N (0: REF/REF, 1: REF/ALT, 2: ALT/ALT), or anti-spike protein IgG levels on day 21 ∼ β_0_ + β_1_∗age + β_2_∗sex + β_3_∗Chinese ethnicity + β_4_∗genotype of Indel N (0: REF/REF, 1: REF/ALT, 2: ALT/ALT). Age, sex, and Chinese ethnicity were used as covariates (Figure S2A). Because the additive model assumes normal distribution, permutation tests with 10,000 permutations were conducted to obtain *p* value without normality assumption. To account for the multiple testing burden, the false discovery rate (FDR) was calculated using the Benjamini–Hochberg procedure and permutation *p* value. An FDR of 10% was used as the cutoff.

#### Reverse transcription and quantitative PCR

Total RNA was isolated from frozen peripheral blood mononuclear cells (PBMCs). One microgram of total RNA was treated with DNase I (Thermo Fisher Scientific, EN0521) to remove genomic DNA contamination, followed by purification using the RNeasy Mini kit (Qiagen, #74106). Subsequently, 100–250 ng of DNase I-treated total RNA was reverse-transcribed using SuperScript III reverse transcriptase and oligo(dT) primers (Thermo Fisher Scientific, #18080051). No-reverse transcriptase (no-RT) controls were included in this step to assess for residual genomic DNA. The resulting cDNA was treated with RNase H at 37°C for 20 min to remove remaining RNA. Quantitative PCR (qPCR) was performed using the Bio-Rad CFX96 Real-Time PCR detection system and SsoAdvanced Universal SYBR Green Supermix (Bio-Rad, #1725275). The primer sequences used for qPCR were as follows: GAPDH forward (F), 5′-CAATGACCCCTTCATTGACC-3’; GAPDH reverse (R), 5′-TTGATTTTGGAGGGATCTCG-3’; SERINC2 forward (F3), 5′-CCTATGCTAGACGCCACACA-3’; SERINC2 reverse (R3), 5′-TCCTGCTCGTTGTCAAAGG-3’; ETFA forward (F1), 5′-GTTTTCTGTCCGTGGAACATCC-3’; ETFA reverse (R1), 5′-ATTTCTGGTCAAGCCACTCTG-3’; ETFA exon 1-2 junction forward (e12juc-F1), 5′-CGTGTGGCTGCAGTAATGGT-3’; ETFA exon 1-2 junction reverse (e12juc-R1), 5′-GCGGCCTCATTGCTACGATT-3’. The quantification cycle (Cq) was determined using the regression mode. Relative gene expression was analyzed using the ΔΔCt method, with *GAPDH* serving as the reference gene. Fold changes in gene expression were calculated relative to a designated reference sample. Samples exhibiting high background signal in the no-RT controls were excluded from further analysis. For *SERINC2*, results from two independent qPCR runs sharing a common reference sample were merged for downstream analysis.

#### Model development using machine learning

nAb levels on day 21 were categorized into low or high antibody responses using an FDA-approved cutoff of 60% inhibition. The quartiles of the age distribution in our mRNA vaccine cohort were also used to categorize individuals into four age groups: <34, 34–51.5, 51.5–66, and >66 years old. To develop a model to predict vaccine-induced antibody responses, the Low/High categorized antibody response was used to represent as the response of the model. 328 individuals were randomly separated into training and test sets (295 and 33 individuals, respectively, see also Figure S5). We first established a base model: vaccine-induced antibody response (Low/High) ∼ age groups. Subsequently, we systematically explored models by incorporating different combinations of sex, Chinese ethnicity, Indel 1 and Indel 3. Models were trained using the BNT162b2 cohort’s training set, with performance optimized through 10-fold cross-validation. The model demonstrating the lowest prediction error rate during cross-validation was selected, and its performance was subsequently assessed on the independent test and Sinopharm sets. The classification Random Forest models were trained on the training set using R package ranger (version 0.17.0).^69^ Grid search was used to tune hyperparameters. For models with different predictors, these hyperparameters were used: num.trees = 2000, min.node.size = 4, replace = T, sample.fraction = 0.25, verbose = F, respect.unordered.factors = “order”. While mtry varied with different models, mtry = 1 for M1 (age), mtry = 2 for M2 (age+sex), mtry = 3 for M3 (age+sex+Chinese ethnicity) and M6 (age+sex+Chinese ethnicity+Indel 1+Indel 3), mtry = 4 for M4 (age+sex+Chinese ethnicity+Indel 1) and M5 (age+sex+Chinese ethnicity+Indel 3). The ROCR R package (version 1.0.11)^70^ was used to plot the ROC curve and calculate the AUC.

#### Surrogate virus neutralization assay

Neutralizing antibodies against SARS-CoV-2 were assessed using the surrogate virus neutralization test (sVNT) platform, following the manufacturer’s protocol (cPass, GenScript).^21^ HRP-conjugated receptor-binding domain (RBD-HRP) was diluted 1:1000 using the provided HRP Dilution Buffer. Plasma samples were diluted 1:10 with Sample Dilution Buffer and then mixed with the diluted RBD-HRP at a 1:1 ratio. The mixtures were incubated at 37 °C for 30 min. Following incubation, 100 μL of each mixture was added to wells of an ACE2-coated plate and incubated at 37 °C for 15 min under a plate seal. The plate was then washed four times with 260 μL of 1× wash buffer to remove unbound RBD-HRP. To detect bound RBD-HRP, 100 μL of 3,3′,5,5′-tetramethylbenzidine (TMB) substrate was added to each well and allowed to develop in the dark for 15 min. The reaction was stopped by adding 50 μL of TMB stop solution. Absorbance at 450 nm was measured using a Cytation 5 microplate reader (BioTek). Percentage inhibition was calculated according to the cPass manual, with a 30% threshold used to define a positive neutralizing antibody response. Further details of the assay are described in Renia et al.^11^

#### Serological detection of anti-spike protein antibodies

A spike protein flow cytometry-based assay was performed to measure anti-SARS-CoV-2 spike protein IgG levels.^22^ Cells expressing the spike (S) protein of the ancestral Wuhan strain, as well as the Delta (B.1.617.2) and Omicron (B.1.1.529, BA.1 subvariant) variants, were utilized in this study. Expression of the respective S proteins was validated using serum from a vaccinated individual who had recovered from a prior COVID-19 infection.^11^ Cells were seeded at a density of 1.5 × 10^5^ cells per well in 96-well V-bottom plates and incubated with human serum diluted 1:100 in 10% FBS. This was followed by a secondary incubation with a dual staining solution containing Alexa Fluor 647-conjugated anti-human IgG (1:500 dilution) and propidium iodide (PI; 1:2500 dilution). Samples were acquired using a BD Biosciences LSR4 laser flow cytometer and analyzed with FlowJo software (version 10, Tree Star). Gating strategies used to identify spike-specific antibody responses are detailed in Goh et al.^22^ The assay was performed in two independent experiments, each with technical duplicates. Further details of the assay are described in Renia et al.^11^

#### Memory B cell ELISpot assay

Memory B cell counts were measured by memory B cell ELISpot assay.^40^ To quantify SARS-CoV-2 RBD-specific memory B cells, the Human IgG (SARS-CoV-2, RBD) ALP ELISpot kit (Mabtech) was employed, following the manufacturer’s guidelines. One million freshly isolated PBMCs were cultured in 1 mL of RPMI medium supplemented with 10% FBS, 1 μg/mL R848, and 10 ng/mL IL-2. These cells were incubated at 37 °C with 5% CO_2_ for 4–5 days to stimulate the differentiation of memory B cells into antibody-producing cells. Following incubation, viable cells were counted, and either 100,000 or 400,000 cells were plated to assess RBD-specific memory B cell responses. To normalize the data, total IgG-secreting cells were measured by plating 1,500 or 3,000 live cells. ELISpot plates were analyzed using the IRIS ELISpot reader (Mabtech), and spot counts were averaged across duplicate wells using the Apex software provided with the IRIS ImmunoSpot system. Further details of the assay are described in Renia et al.^11^

#### Pseudovirus neutralization assays

The neutralization assay using pseudotyped lentiviruses was carried out as previously reported.^11^ For the assay, CHO cells stably expressing human ACE2 (CHO-ACE2), generously provided by Professor Yee-Joo Tan (Department of Microbiology, NUS and IMCB, A∗STAR, Singapore) were utilized. These cells were seeded at a density of 1.8 × 10^4^ cells per well in 96-well black microplates (Corning) and cultured overnight in medium without Geneticin. Heat-inactivated plasma samples were serially diluted from 1:5 to 1:5120 in 4-fold steps and incubated with an equal volume of pseudovirus bearing the SARS-CoV-2 spike protein from either the ancestral strain, Delta variant, or Omicron variant (5 ng p24 per well) at 37 °C for 1 h. The virus-plasma mixtures were then added to the pre-seeded CHO-ACE2 cells in duplicate wells. After a 1-h incubation, the medium was refreshed. Following a 48-h incubation period, cells were washed with PBS and lysed using 1× Passive Lysis Buffer (Promega) with gentle shaking at 125 rpm for 30 min at 37 °C. Luciferase activity was measured using the Luciferase Assay System (Promega) on a GloMax Luminometer (Promega). Further details of the assay are described in Renia et al.^11^

### Quantification and statistical analysis

Variance was calculated using R version 4.3.1 (Figure 1B and 1C). To assess the association between antibody responses and infection outcomes, infections were recorded for 201 participants in the cohort over a one-year period following vaccination. Logistic regression analysis was conducted to evaluate the relationship between infection outcomes and nAb levels or anti-spike protein IgG levels on days 21, 90, and 180 (Figure 1D and 1E). Linear regression was used to examine the association between nAb levels on day 21 and demographic factors including age, sex, and Chinese ethnicity. Similarly, linear regression was applied to assess the relationship between anti-spike protein IgG levels on day 21 and the same demographic variables. Multiple linear regression analyses were performed to further investigate the association of nAb and anti-spike protein IgG levels on day 21 with combinations of age and sex, as well as age, sex, and Chinese ethnicity (Figure 2D). The association of indels with different traits were tested using the following additive models: memory B-cell counts on day 360 ∼ β_0_ + β_1_∗genotype of Indel N (0: REF/REF, 1: REF/ALT, 2: ALT/ALT) (Figure 3B), pseudovirus-neutralization activities for Delta or Omicron strains on day 90 ∼ β_0_ + β_1_∗genotype of Indel N (0: REF/REF, 1: REF/ALT, 2: ALT/ALT) (Figure 3D), nAb levels on day 90 in the Sinopharm cohort ∼ β_0_ + β_1_∗Age +β_2_∗Sex +β_3_∗Chinese ethnicity +β_4_∗genotype of Indel N (0: REF/REF, 1: REF/ALT, 2: ALT/ALT) (Figure 4C), Foldchange of SERINC2 polyA + RNAs ∼ β_0_ + β_1_∗genotype of Indel N (0: REF/REF, 1: REF/ALT, 2: ALT/ALT) (Figure S3E), Foldchange of ETFA polyA + RNAs ∼ β_0_ + β_1_∗genotype of Indel N (0: REF/REF, 1: REF/ALT, 2: ALT/ALT) (Figure S4E), Foldchange of ETFA exon2+ polyA + RNAs ∼ β_0_ + β_1_∗genotype of Indel N (0: REF/REF, 1: REF/ALT, 2: ALT/ALT) (Figure S4E). R version 4.3.1^71^ was utilized to conduct these tests, with a significance level set at a *p* value of 0.05. The association between indels and vaccine-induced antibody responses for participants in the BNT162b2 cohort were detailed in the section of “Association between indels and antibody responses.”

## Supplemental information

Document S1. Figures S1–S5

Table S1. Source data supporting key findings in the study, related to Table 1, Figure 1, 2, 3, 4 and S1
