# Supplementary material for: Human indels as predictors of antibody responses to COVID-19 vaccines
Source: iScience. 2025 Sep 1;28(10):113475. doi: 10.1016/j.isci.2025.113475 (PMC12570367; doi:10.1016/j.isci.2025.113475)
Supplement: Document S1. Figures S1–S5 [file mmc1.pdf]

iScience, Volume *28*

Supplemental information

Human indels as predictors of antibody responses to COVID-19 vaccines

Hsiuyi V. Chen, Siew-Wai Fong, Yun Shan Goh, Matthew Zirui Tay, Angeline Rouers, Zi Wei Chang, Andrea Wei Ming Chua, Liang Hui Loo, SCOPE cohort Study Group, Jean-Marc Chavatte, Raymond Tzer Pin Lin, Yee-Sin Leo, Chiea Chuen Khor, David C. Lye, Laurent Renia, Barnaby Edward Young, and Lisa F.P. Ng

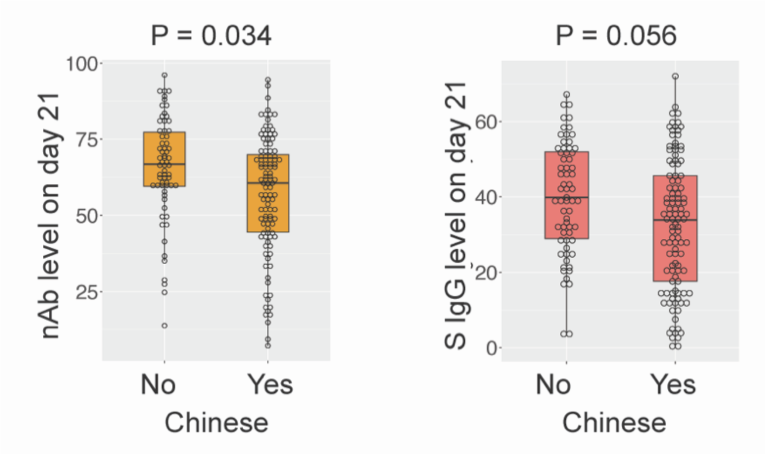

Figure S1. Effects of Chinese ethnicity on neutralizing antibody and anti-spike protein antibody responses in individuals without reported pre-existing conditions.
Among individuals without reported pre-existing conditions (Supplementary Table 1_Pre-existing conditions), Chinese ethnicity was significantly associated with nAb levels on day 21 (linear regression, adjusted for age, p=0.034), but not with anti-spike protein IgG levels on day 21 (adjusted for age, p=0.056). The boxplots show the median and interquartile range of the data.

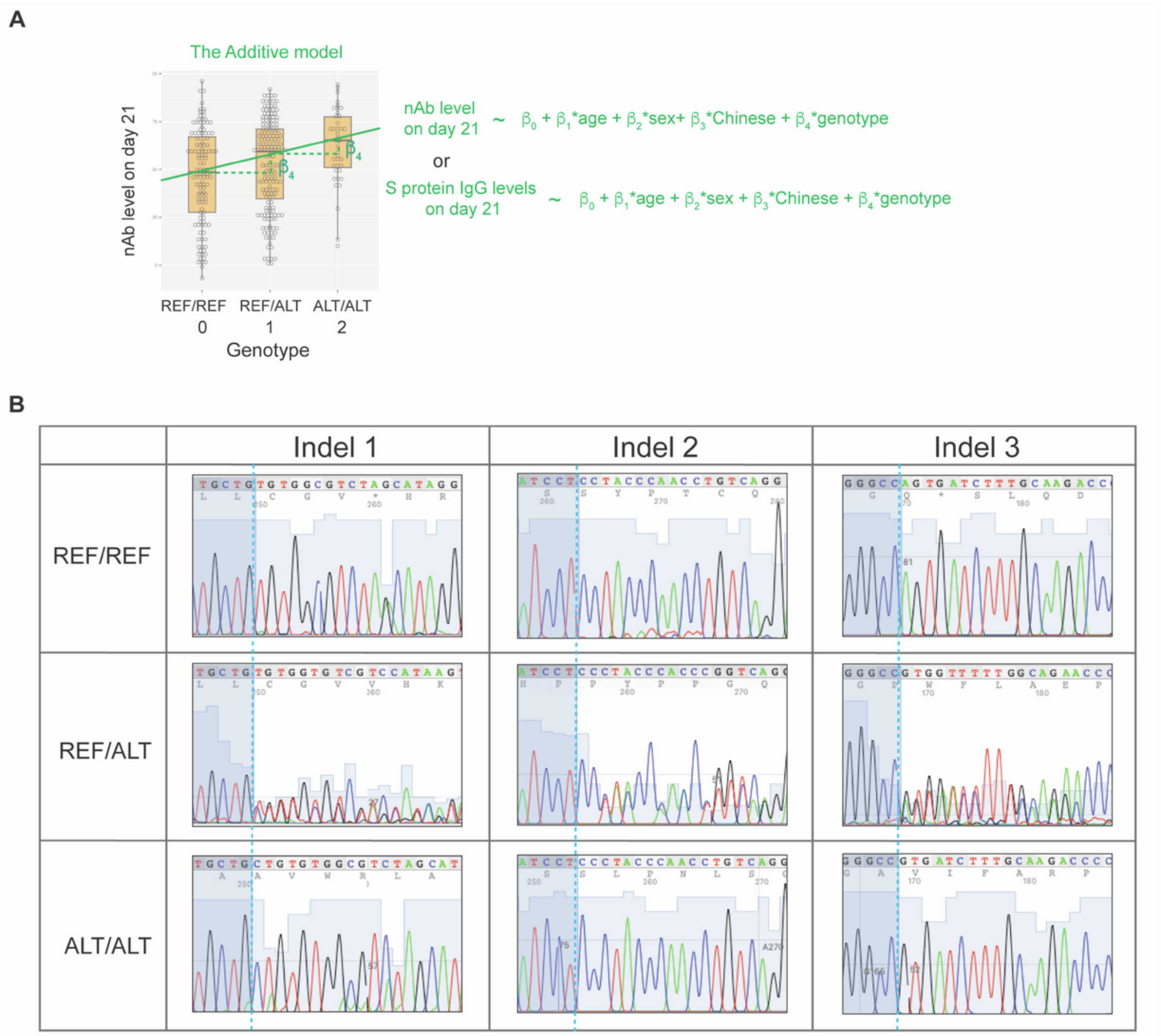

Figure S2. Additive model and validation of indels by Sanger sequencing.

(**A**) Illustration of the additive model of nAb levels on day 21 ~ $\beta_0 + \beta_1 \times$ age + $\beta_2 \times$ sex + $\beta_3 \times$ Chinese ethnicity + $\beta_4 \times$ genotype of Indel N (0: REF/REF, 1: REF/ALT, 2: ALT/ALT), or anti-spike protein IgG levels on day 21 ~ $\beta_0 + \beta_1 \times$ age + $\beta_2 \times$ sex + $\beta_3 \times$ Chinese ethnicity + $\beta_4 \times$ Indel N (0: REF/REF, 1: REF/ALT, 2: ALT/ALT), using age, sex, and Chinese ethnicity as covariates. (**B**) Sanger sequencing results for indels 1-3. For each indel, blood samples from 15 individuals with different genotypes (REF/REF, REF/ ALT, or ALT/ALT) from the COVID-19 mRNA vaccine cohort were analysed by Sanger sequencing. The results from 15 individuals confirmed the genotypes of indels 1-3 from whole-exome sequencing analysis.

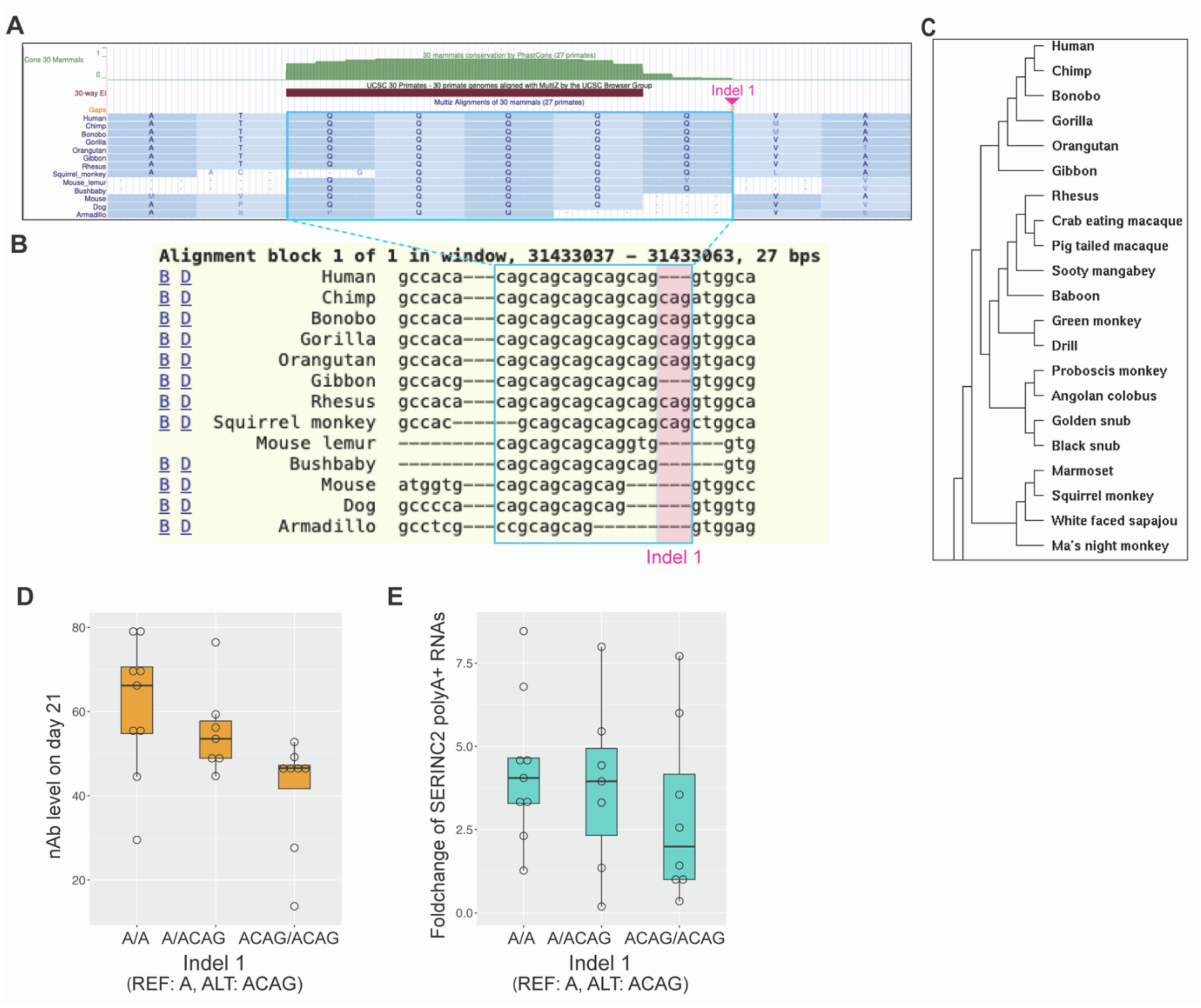

Figure S3. Evolutionary Conservation of Indel 1 and its impact on *SERINC2* expression. (**A**) Evolutionary conservation at Indel 1 locus. UCSC Genome Browser tracks depicting multi-species sequence alignment and evolutionary conservation score for the Indel 1 locus across 30 mammals. **(B)** DNA sequence alignment of Indel 1 in primates and other mammalian species. **(C)** A partial phylogenetic tree representing the evolutionary relationships of the 30 mammalian species used to assess conservation at Indel 1. **(D)** Distribution of nAb levels on day 21 across different Indel 1 genotypes in 24 selected individuals from the BNT162b2 cohort. **(E)** Effect of Indel 1 on the expression levels of *SERINC2* polyA+ RNA. Expression levels were displayed as fold changes relative to a designated reference sample. For (D) and (E), the boxplots show the median and interquartile range of the data.

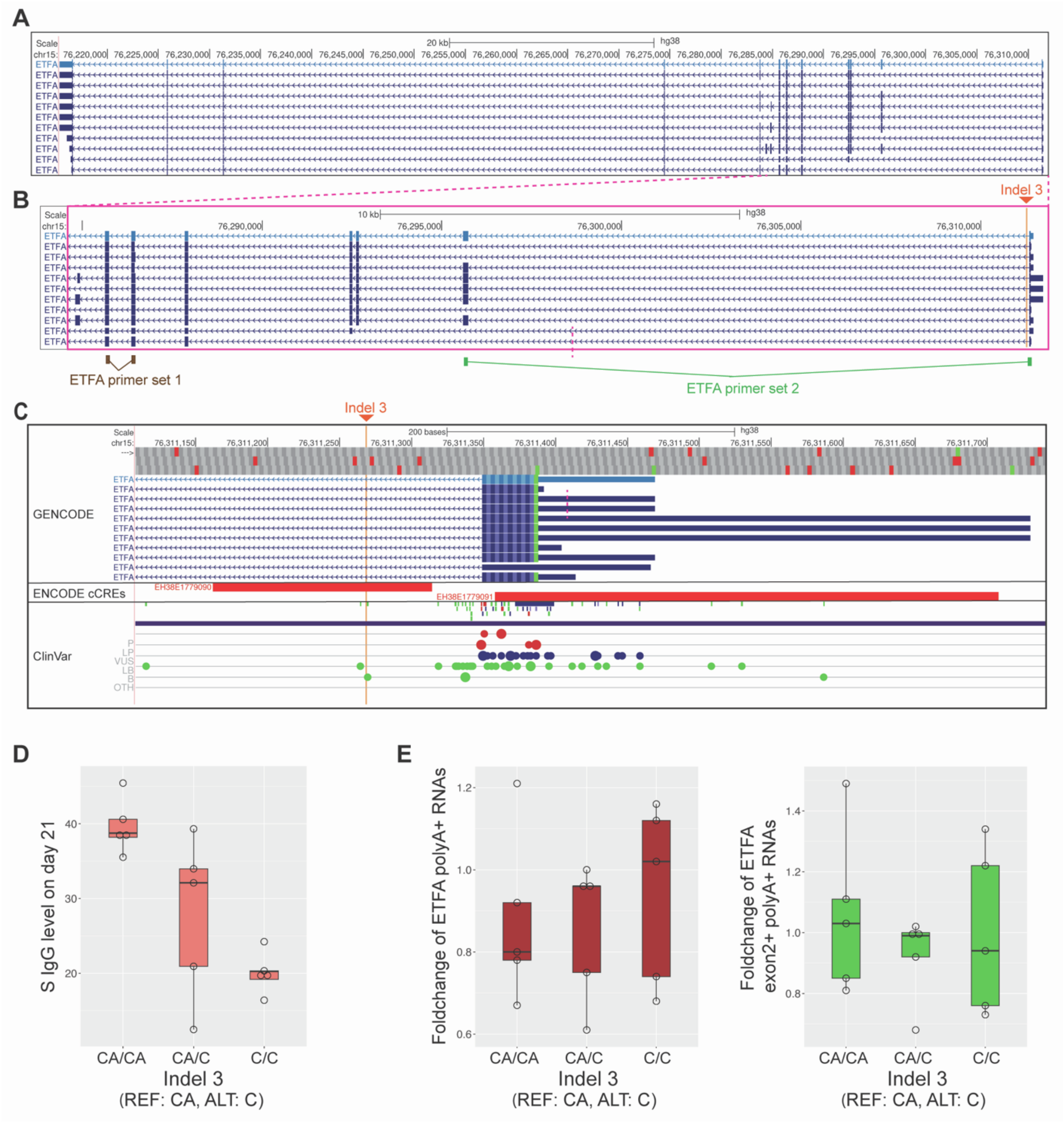

Figure S4. Effect of Indel 3 on *ETFA* gene splicing and isoform levels.

(A) *ETFA* gene's 11 splicing isoforms, as displayed in the GENCODE UCSC Genome Browser track. **(B)** Magnified view of the *ETFA* region from exon 1 to exon 8. The location of Indel 3 in intron 1 is indicated in orange. Target exons of the two primers sets, which amplify exon-exon junctions, are shown in pairs: brown for exon 6-7 and green for exon 1-2. **(C)** UCSC Genome Browser tracks illustrating Indel 3's location within a potential promoter element, as identified by the ENCODE project, and its cataloging in the ClinVar database. **(D)** Distribution of anti-spike protein IgG levels on day 21 across different Indel 3 genotypes in 15 selected individuals from the BNT162b2 cohort. **(E)** Effect of Indel 3 on *ETFA* polyA+ RNA expression levels. Expression levels are presented as fold changes relative to a designated reference sample. The left panel shows total *ETFA* polyA+ RNA transcript levels measured by *ETFA* primer set 1; the right panel shows *ETFA* exon2+ polyA+ RNA transcript levels measured by *ETFA* primer set 2. For (D) and (E), the boxplots show the median and interquartile range of the data.

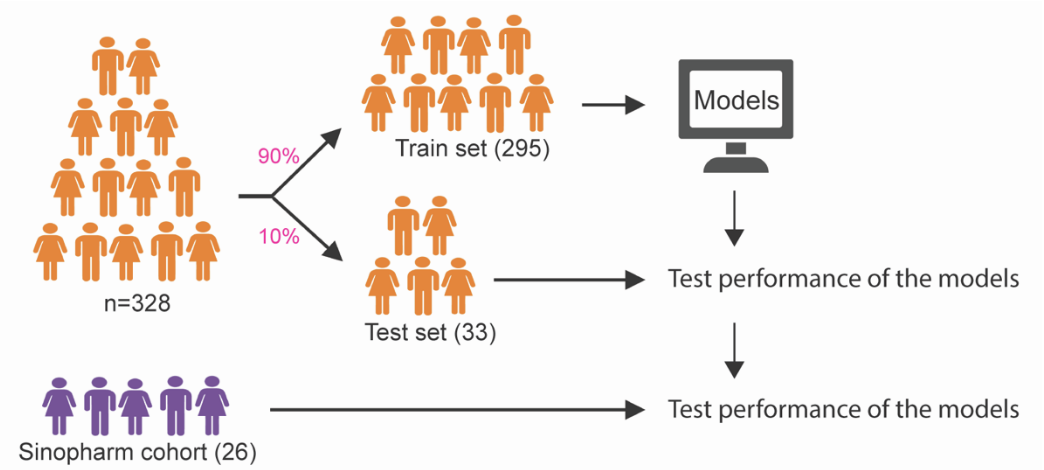

Figure S5. Modelling strategy.
Individuals from the COVID-19 mRNA BNT162b2 vaccine cohort (n=328) were randomly divided into training and test sets comprising 295 and 33 individuals, respectively. Predictive models were trained using the training set. The performance of the models was tested with the test set and the Sinopharm cohort data.
